# The association between sialolithiasis and smoking, alcohol drinking and obesity in Korea: a nested case-control study

**DOI:** 10.1186/s12889-020-08674-w

**Published:** 2020-04-17

**Authors:** Young Ju Jin, Young Eun Han, Hyo Geun Choi

**Affiliations:** 1 Department of Otorhinolaryngology-Head & Neck Surgery, Wonkwang University Hospital, Wonkwang University College of Medicine, Iksan, South Korea; 2 Department of Otorhinolaryngology-Head and Neck Surgery, Seoul National University College of Medicine, Seoul, South Korea; 3 Department of Otorhinolaryngology-Head & Neck Surgery, Hallym University College of Medicine, Anyang, South Korea; 4 Department of Otorhinolaryngology-Head & Neck Surgery, Hallym University Sacred Heart Hospital, 22, Gwanpyeong-ro 170beon-gil, Dongan-gu, Anyang-si, Gyeonggi-do 14068 Republic of Korea

**Keywords:** Sialolithiasis, Smoker, Alcohol, Obesity, Nested case-control study

## Abstract

**Background:**

Smoking and alcohol consumption are the most common social habits in patients with sialolithiasis. Moreover, obesity has been reported to have a significant association with poor oral hygiene, one of the causes of sialolithiasis. The purpose of this study was to evaluate the relationships among tobacco smoking, drinking alcohol, obesity and sialolithiasis in a Korean population.

**Methods:**

The Korean National Health Insurance Service-Health Screening Cohort, which includes patients ≥40 years old, was assessed from 2002 to 2013. A total of 947 sialolithiasis participants were matched with 3788 control subjects at a ratio of 1:4 with respect to age group, sex, income group, region of residence, hypertension, diabetes, and dyslipidemia. We analyzed the participants’ previous histories of smoking (current or past smokers compared to nonsmokers) and alcohol consumption (≥ 1 time per week compared to < 1 time per week) in the sialolithiasis and control groups. Obesity was measured using body mass index (BMI, kg/m^2^), which was categorized as < 18.5 (underweight), ≥ 18.5 and < 23 (normal), ≥ 23 and < 25 (overweight), ≥ 25 and < 30 (obese I), and ≥ 30 (obese II). Crude and adjusted odds ratios (ORs) and 95% confidence intervals (CIs) were calculated using conditional logistic regression analyses.

**Results:**

The rate of smoking was higher in the sialolithiasis group (32.4% [307/947]) than in the control group (29.1% [1103/3788], *P* = 0.047). The adjusted OR of smoking for the sialolithiasis group was 1.31 (95% CI = 1.08–1.59, *P* = 0.006). Alcohol consumption and obesity were not statistically significantly related to sialolithiasis.

**Conclusion:**

The odds of smoking were increased in sialolithiasis patients compared with control subjects in the population ≥ 40 years of age.

## Background

Sialolithiasis, which is the presence of a salivary duct stone, is the most common cause of major salivary gland swelling and pain in the ipsilateral salivary gland, typically when eating [1, 2]. The annual incidence of sialolithiasis was 5.5/100,000 in Denmark in 2015 [3]; 2.7–5.9/100,000 in England in 1999 and 2000 [4, 5], based on hospital data; and 1.4–2.3/100,000 in Taiwan from 1996 to 2013, based on a health insurance research database [6]. The incidence rate increased to 7.3–14.1/100,000 from 2.05–3.98/100,000 in children (those younger than 20 years old) in a Danish population-based study [7]. However, there are no reports on the incidence rate of sialolithiasis in Korea. The submandibular glands are the most commonly affected glands (80–90%), followed by the parotid glands (5–20%) and the sublingual glands (very rare) [1, 2, 8]. The predominant prevalence in the submandibular gland occurs because Wharton’s duct has a longer course and a larger diameter than Stenson’s duct; thus, saliva flows against gravity and has a more viscous composition with a higher calcium and mucin content in this gland than in other salivary glands [9, 10].

The exact pathogenesis of stone formation in the salivary gland remains unclear. It is generally accepted that reduced saliva flow leads to an accumulation of salivary stones [11]. The retrograde theory suggests that food substances or bacteria in the oral cavity may ascend the salivary ducts and act as a nidus for further calcification [12].

Smoking and alcohol consumption are the most common social habits in patients with sialolithiasis. The association between sialolithiasis and smoking has been reported in several previous studies [1, 10, 11, 13, 14]. However, evidence of an association between alcohol consumption and sialolithiasis is lacking even though alcohol drinking is one of the most common causes of oral cavity and deep neck infection [15, 16]. Obesity has been reported as a risk factor for various kinds of inflammatory disease, including periodontal disease [17]. In addition, previous studies have reported that poor oral health behaviors showed positive correlation with obesity [18, 19]. Therefore, we hypothesized that obesity could be related to sialolithiasis caused by an oral infection that spreads to a salivary duct. The aim of this study was to evaluate the association between tobacco smoking, alcohol consumption, and obesity and sialolithiasis in a nationwide population-based cohort study using data from the Korean National Health Insurance Service-Health Screening Cohort (NHIS-HEALS). In this study, we estimated the odds ratios (ORs) of smoking, alcohol consumption and obesity in sialolithiasis patients compared to participants in a 1:4 matched control group.

## Materials and methods

### Study population and data collection

This national cohort study relied on data from the Korean NHIS-HEALS, previously used in several peer reviewed publications [20–23]. The ethics committee of Hallym University (2017-I102) approved the use of these data. The NHIS provides a representative sample database with a substantial volume of representative information that does not require privacy regulation for public health research and policy development. Therefore, the data used in our research is allowed to be made publicly available. In this reason, written informed consent was exempted by the Institutional Review Board.

The Korean NHIS selects random samples of ~ 10% (*n* = ~ 515,000) directly from the entire population that underwent health evaluations from 2002 through 2013 (*n* = ~ 5,150,000). Age- and sex-specific distributions of the cohort population have been described online [24]. The details of the methods used to perform these procedures are provided by the National Health Insurance Sharing Service [25].

All insured Koreans who are at least 40 years old and their dependents undergo no-cost biannual health evaluations. Each examinee must complete a standard questionnaire for this health evaluation program [26]. Because all Korean citizens are recognized by a 13-digit resident registration number from birth to death, exact population statistics can be determined using this database and the duplication of records can be identified. It is mandatory for all Koreans to enroll in the NHIS. Moreover, all medical treatments in Korea can be tracked without exception using the Korean Health Insurance Review & Assessment (HIRA) system. In Korea, it is legally required to provide notice of death to an administrative entity before the funeral and the causes and date of death are recorded by medical doctors on a death certificate.

This cohort database included (i) personal information, (ii) health insurance claim codes (procedures and prescriptions), (iii) diagnostic codes based on the International Classification of Disease-10 (ICD-10), (iv) death records from the Korean National Statistical Office (using the Korean Standard Classification of disease), (v) socioeconomic data (residence and income), (vi) medical examination data, and (vii) health check-up data (body mass index [BMI], alcohol consumption, smoking habits, blood pressure, urinalysis, hemoglobin, fasting glucose, lipid parameters, creatinine, and liver enzymes) for each participant for the period from 2002 to 2013 [25, 26]. Alcohol consumption and smoking habits were surveyed, and BMI was calculated by measuring height and weight at health check-ups.

### Participant selection

**Fig. 1:**
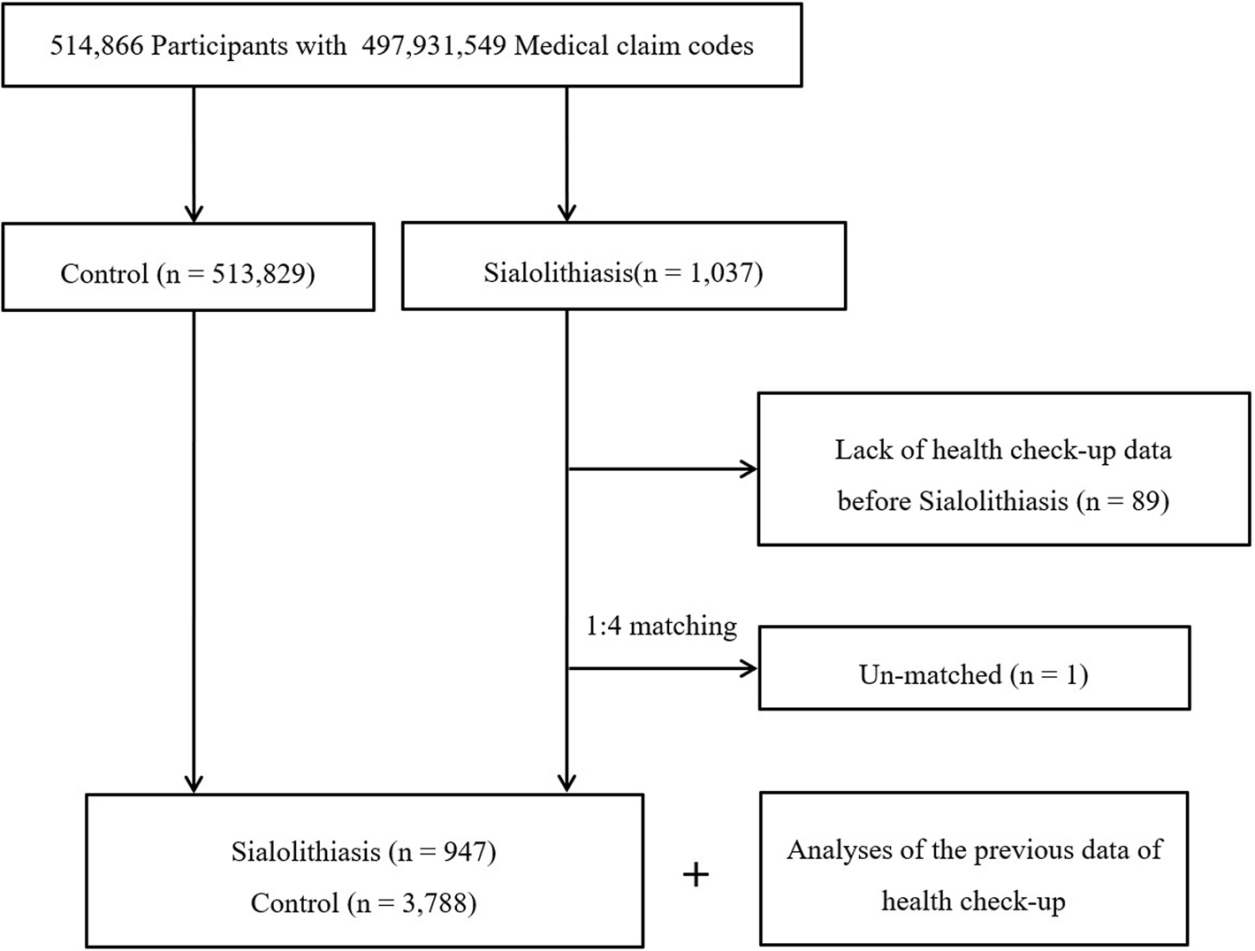
Schematic illustration of the participant selection process used in the present study. Of a total of 514,866 participants, 947 sialolithiasis patients were matched with 3788 control participants by age group, sex, income group, region of residence, and past medical history

Out of 514,866 cases with 497,931,549 medical claim codes, we included participants who were diagnosed with sialolithiasis (*n* = 1037). The sialolithiasis participants were matched 1:4 with participants among this cohort who were never diagnosed with sialolithiasis from 2002 through 2013 (control group). The control group was selected from the original population (*n* = 513,829). Subjects were matched by age group, sex, income group, region of residence, and medical history (e.g., hypertension, diabetes, and dyslipidemia). Participants in the control group were sorted using a random number order and selected from top to bottom to prevent selection bias. It was assumed that the matched control participants were involved at the same time as each matched participant with sialolithiasis (index date). Therefore, participants in the control group who died before the index date were excluded. Participants with sialolithiasis who had no history of health evaluations before the index date were excluded (*n* = 89). One participant with sialolithiasis was excluded due to the lack of a matching participant. Finally, 1:4 matching resulted in the inclusion of 947 participants with sialolithiasis and 3788 control participants. We analyzed the previous health evaluation data in the sialolithiasis and control groups after matching (Fig. 1). In this study, we used the most recent health evaluation data before the index date.

### Variables

#### Independent variables

Tobacco smoking was categorized based on the current smoking status (nonsmoker or past smoker/current smoker), duration of smoking (nonsmoker, < 20 years, and ≥ 20 years), and current number of cigarettes smoked per day (0 cigarettes per day, < 20 cigarettes per day, and ≥ 20 cigarettes per day, S1 Table). We selected the current smoking status in this study. Current and past smokers were defined as smokers and were compared to nonsmokers.

Alcohol consumption was evaluated by frequency (< 1 time per week, and ≥ 1 time per week) and by the amount of alcohol consumed at a time (< 1 soju bottle, 1 soju bottle, and > 1 soju bottle, S1 Table). Generally, a bottle of soju contains 17.5% of alcohol per 360 ml. A bottle of soju is equivalent to approximately 3.5 bottles of beer. We selected the frequency of alcohol consumption in this study. We used alcohol consumption to define alcohol consumption ≥1 time per week compared to alcohol consumption < 1 time per week.

Obesity was measured using BMI (kg/m^2^) and was categorized as < 18.5 (underweight), ≥ 18.5 and < 23 (normal), ≥ 23 and < 25 (overweight), ≥ 25 and < 30 (obese I), and ≥ 30 (obese II) following the WPRO 2000 guidelines [27].

#### Covariate analysis

The age groups were classified using 5-year age intervals: 40–44, 45–49, 50–54, …, and 85+ years old. A total of 10 age groups were designated. Income was initially divided into 41 classes according to health care premium (one health assistance class, 20 self-employment health insurance classes, and 20 employment health insurance classes). These groups were recategorized into 5 classes (classes 1 [lowest income]-5 [highest income]). The region of residence was divided into 16 areas according to administrative district. These regions were regrouped into urban (Seoul, Busan, Daegu, Incheon, Gwangju, Daejeon, and Ulsan) and rural (Gyeonggi, Gangwon, Chungcheongbuk, Chungcheongnam, Jeollabuk, Jeollanam, Gyeongsangbuk, Gyeongsangnam, and Jeju) areas.

The participants’ prior medical histories were evaluated using ICD-10 codes. To ensure an accurate diagnosis, hypertension (I10 and I15), diabetes (E10-E14), and dyslipidemia (E78) were regarded as present if a participant was treated ≥2 times.

#### Dependent variable

Sialolithiasis was diagnosed based on the ICD-10 code K115.

### Statistical analyses

Chi-square tests were used to compare the general characteristics of the sialolithiasis and control groups.

To analyze the ORs of smoking, drinking alcohol, and obesity in sialolithiasis patients, conditional logistic regression analysis was used. In this analysis, a crude (simple) adjusted model (adjusted for obesity, smoking status, and frequency of alcohol consumption) was used, and 95% confidence intervals (CIs) were calculated. In these analyses, age group, sex, income group, region of residence, hypertension, diabetes, and dyslipidemia were stratified.

For the subgroup analyses, we divided the participants by age and sex (< 60 years, ≥ 60 years; male and female). The division of the age groups was determined by the median age of all the participants.

Two-tailed analyses were conducted, and *P* values less than 0.05 were considered to indicate significance. The results were statistically analyzed using SPSS v. 22.0 (IBM, Armonk, NY, USA).

## Results

The rate of tobacco smoking was higher in the sialolithiasis group (32.4% [307/947]) than in the control group (29.1% [1103/3788], *P* = 0.047, Table 1). Alcohol consumption and obesity were not different between the sialolithiasis and control groups. The general characteristics (age, sex, income, region of residence, hypertension, diabetes, and dyslipidemia) of the participants were the same due to the matching procedure (*P* = 1.000).

**Table 1:** General characteristics of the participants

Characteristics	Participants		
	Sialolithiasis (n, %)	Control (n, %)	*P*-value
Age (years)			1.000
40–44	36 (3.8)	144 (3.8)	
45–49	135 (14.3)	540 (14.3)	
50–54	215 (22.7)	860 (22.7)	
55–59	172 (18.2)	688 (18.2)	
60–64	144 (15.2)	576 (15.2)	
65–69	102 (10.8)	408 (10.8)	
70–74	88 (9.3)	352 (9.3)	
75–79	33 (3.5)	132 (3.5)	
80–84	19 (2.0)	76 (2.0)	
85+	3 (0.3)	12 (0.3)	
Sex			1.000
Male	493 (52.1)	1972 (52.1)	
Female	454 (47.9)	1816 (47.9)	
Income			1.000
1 (lowest)	130 (13.7)	520 (13.7)	
2	100 (10.6)	400 (10.6)	
3	128 (13.5)	512 (13.5)	
4	222 (23.4)	888 (23.4)	
5 (highest)	367 (38.8)	1468 (38.8)	
Region of residence			1.000
Urban	408 (43.1)	1632 (43.1)	
Rural	539 (56.9)	2156 (56.9)	
Hypertension	290 (30.6)	1160 (30.6)	1.000
Diabetes	117 (12.4)	468 (12.4)	1.000
Dyslipidemia	105 (11.1)	420 (11.1)	1.000
Obesity (BMI, kg/m^2^)			0.100
< 18.5	13 (1.4)	92 (2.4)	
≥ 18.5 to < 23	314 (33.2)	1272 (33.6)	
≥ 23 to < 25	280 (29.6)	1051 (27.7)	
≥ 25 to < 30	299 (31.6)	1250 (33.0)	
≥ 30	41 (4.3)	123 (3.2)	
Smoking			0.047*
Nonsmoker	640 (67.6)	2685 (70.9)	
Past or current smoker	307 (32.4)	1103 (29.1)	
Alcohol consumption			0.334
< 1 time per week	717 (75.7)	2810 (74.2)	
≥ 1 time per week	230 (24.3)	978 (25.8)	

BMI: body mass index, kg/m^2^

*Chi-square test. Significance at *P* < 0.05

The adjusted OR for smoking in the sialolithiasis group was 1.31 (95% CI = 1.08–1.59, *P* = 0.006, Table 2). The adjusted ORs for alcohol consumption and obesity in the sialolithiasis group did not reach statistical significance. The adjusted OR for alcohol consumption in the sialolithiasis group was 0.86 (95% CI = 0.71–1.04, *P* = 0.119). The adjusted ORs for obesity in the sialolithiasis group were 0.56 (95% CI = 0.31–1.01, *P* = 0.055) for BMI < 18.5; 1.00 for BMI ≥ 18.5 to < 23; 1.09 (95% CI = 0.90–1.31, *P* = 0.376) for BMI ≥ 23 to < 25; 0.98 (95% CI = 0.81–1.17, *P* = 0.800) for BMI ≥ 25 to < 30; and 1.36 (95% CI = 0.93–1.98, *P* = 0.119) for BMI ≥ 30.

**Table 2:** Crude and adjusted odds ratios (95% confidence interval) for smoking, drinking alcohol, and obesity in sialolithiasis patients

Characteristics	Crude model		Adjusted model	
	Crude†	*P*-value	Adjusted†‡	*P*-value
Smoking	1.27 (1.05–1.53)	0.016*	1.31 (1.08–1.59)	0.006*
Alcohol	0.90 (0.75–1.09)	0.281	0.86 (0.71–1.04)	0.119
Obesity (BMI, kg/m^2^)		0.100		0.092
< 18.5	0.57 (0.31–1.03)	0.063	0.56 (0.31–1.01)	0.055
≥ 18.5 to < 23	1.00		1.00	
≥ 23 to < 25	1.08 (0.90–1.30)	0.395	1.09 (0.90–1.31)	0.376
≥ 25 to < 30	0.97 (0.81–1.17)	0.757	0.98 (0.81–1.17)	0.800
≥ 30	1.36 (0.93–1.98)	0.111	1.36 (0.93–1.98)	0.119

* Conditional logistic regression analysis, significance at *P* < 0.05

† Conditional model for obesity, smoking status (current or past smoker compared to nonsmoker), and frequency of alcohol consumption (≥ 1 time per week compared to < 1 time per week)

‡ Adjusted model including obesity, smoking status and frequency of alcohol consumption

In subgroup analyses performed according to age and sex, the adjusted OR for smoking with statistical significance was 1.36 and 1.27 for participants < 60 years old (95% CI = 1.06–1.75, *P* = 0.017) and for men (95% CI = 1.03–1.56, *P* = 0.023, Table 3), respectively. The crude and adjusted ORs for drinking alcohol and obesity were not statistically significant for either sex and all age groups. The rates of smoking, alcohol consumption, and obesity were evaluated according to age groups (< 60 years vs. ≥60 years) and sex (male vs. female). The rates were significantly different among all the groups (S2).

**Table 3:** Crude and adjusted odds ratios (95% confidence interval) for smoking, drinking alcohol, and obesity in sialolithiasis patients in each stratified group according age and sex

Characteristics	Crude model		Adjusted model	
	Crude†	P-value	Adjusted†‡	*P*-value
**< 60 years (*n*** **= 2790)**				
Smoking	1.32 (1.03–1.69)	0.027*	1.36 (1.06–1.75)	0.017*
Alcohol consumption	0.95 (0.75–1.19)	0.628	0.90 (0.71–1.13)	0.352
Obesity (BMI, kg/m^2^)		0.164		0.164
< 18.5	0.65 (0.29–1.48)	0.307	0.63 (0.28–1.43)	0.269
≥ 18.5 to < 23	1.00		1.00	
≥ 23 to < 25	0.96 (0.76–1.22)	0.761	0.96 (0.76–1.22)	0.748
≥ 25 to < 30	0.87 (0.69–1.10)	0.253	0.87 (0.69–1.11)	0.256
≥ 30	1.50 (0.95–2.39)	0.084	1.49 (0.94–2.36)	0.092
**≥ 60 years (*n*** **= 1945)**				
Smoking	1.19 (0.88–1.61)	0.266	1.24 (0.91–1.69)	0.168
Alcohol consumption	0.83 (0.61–1.14)	0.244	0.80 (0.58–1.10)	0.171
Obesity (BMI, kg/m^2^)		0.178		0.160
< 18.5	0.52 (0.22–1.24)	0.141	0.52 (0.22–1.23)	0.137
≥ 18.5 to < 23	1.00		1.00	
≥ 23 to < 25	1.30 (0.97–1.75)	0.079	1.32 (0.98–1.77)	0.070
≥ 25 to < 30	1.15 (0.86–1.54)	0.340	1.16 (0.87–1.55)	0.313
≥ 30	1.11 (0.57–2.16)	0.756	1.13 (0.58–2.20)	0.724
**Male (*n*** **= 2645)**				
Smoking	1.24 (1.02–1.52)	0.034*	1.27 (1.03–1.56)	0.023*
Alcohol consumption	0.94 (0.77–1.14)	0.514	0.89 (0.73–1.10)	0.284
Obesity (BMI, kg/m^2^)		0.142		0.144
< 18.5	0.47 (0.21–1.07)	0.071	0.47 (0.21–1.06)	0.068
≥ 18.5 to < 23	1.00		1.00	
≥ 23 to < 25	0.99 (0.77–1.28)	0.940	0.99 (0.77–1.28)	0.962
≥ 25 to < 30	0.85 (0.66–1.10)	0.221	0.86 (0.66–1.11)	0.233
≥ 30	1.38 (0.79–2.41)	0.259	1.37 (0.79–2.40)	0.265
**Female (*n*** **= 2270)**				
Smoking	1.47 (0.83–2.58)	0.186	1.68 (0.94–3.00)	0.082
Alcohol consumption	0.75 (0.47–1.21)	0.243	0.69 (0.42–1.13)	0.136
Obesity (BMI, kg/m^2^)		0.505		0.435
< 18.5	0.70 (0.29–1.69)	0.427	0.66 (0.28–1.60)	0.361
≥ 18.5 to < 23	1.00		1.00	
≥ 23 to < 25	1.18 (0.91–1.54)	0.220	1.19 (0.91–1.55)	0.205
≥ 25 to < 30	1.11 (0.86–1.45)	0.421	1.13 (0.87–1.47)	0.367
≥ 30	1.36 (0.81–2.27)	0.241	1.37 (0.82–2.30)	0.225

* Conditional logistic regression analysis, significance at *P* < 0.05

† Conditional model including age, sex, income, region of residence, hypertension, diabetes, and dyslipidemia history

‡ Model adjusted for obesity, smoking status (current or past smoker vs. nonsmoker), and frequency of alcohol consumption (≥ 1 time per week compared to < 1 time per week)

Additionally, the association between smoking duration and sialolithiasis was evaluated. The adjusted ORs for the association between smoking and sialolithiasis were 1.34 (95% CI = 1.08–1.66, *P* = 0.007 among those with a duration of smoking ≥20 years and 1.24 (95% CI = 0.94–1.63, *P* = 0.123, S3 table) among those with a duration of smoking < 20 years.

## Discussion

In our study, the rate of smoking was higher in the sialolithiasis group (32.4%) than in the control group (29.1%), and the adjusted OR of smoking was significantly higher in the sialolithiasis group than in the control group (adjusted OR = 1.31, 95% CI = 1.08–1.59). However, the rates of alcohol consumption and obesity did not differ between the sialolithiasis and control groups. We found the similar results when we analyzed these factors according to the duration of smoking and the amount of alcohol consumed. In particular, longer periods of smoking were significantly related to sialolithiasis. Similar to our findings, a history of smoking has been reported to be a predisposing factor for sialolithiasis in the general population (adjusted OR = 1.21, 95% CI = 1.02–1.44, *P* = 0.028) [28]. Stone sizes have been reported to be much larger in current smokers than in ex-smokers (12.4 mm vs. 7.5 mm, *P* = 0.03) [13].

Tobacco smoking can cause oral cavity inflammation [29]. Because the salivary duct opens to the oral cavity, inflammation in the oral cavity can be transmitted into the salivary duct and cause ductal swelling and narrowing. Decreased salivary duct diameter can induce salivary stasis and cause the precipitation of salivary stones. Salivary stones and ductal inflammation increase the occurrence of sialolithiasis. After the formation of salivary duct stones, bacteria can ascend the duct more easily from the mouth and proliferate on the surface of the stones, contributing to the further development of sialolithiasis [11]. Moreover, long-term smoking decreases salivary flow and thus decreases antimicrobial proteins, including IgA, a-amylase and lysozyme, which are important for oral mucosal immunity [30, 31]. Therefore, a vicious cycle that accelerates the formation of salivary gland stones ensues.

In this study, alcohol consumption and obesity did not differ between the sialolithiasis and control groups. Similar to tobacco smoking, alcohol consumption and obesity play an important role in oral health. There was a study that found no significant difference in alcohol consumption between the 50 cases with salivary gland stones and the 90 cases without stones (*P* = 0.597) [32]. Because sialolithiasis can be promoted by oral cavity inflammation, we presumed that alcohol intake might be associated with sialolithiasis. Generally, the association between alcohol consumption and inflammation is known to depend on the amount of alcohol consumed. The influence of low to moderate alcohol intake on health outcomes is controversial; however, excessive alcohol intake is associated with worse outcomes [33]. In particular, light to moderate drinking has shown protective effects against cardiovascular disease, and this amount has been defined as daily consumption of a 5 to 6 oz. glass of red wine [34]. In our data, we did not compare the amounts of alcohol consumed; instead, we examined the frequency of alcohol consumption (< 1 time per week, ≥ 1 time per week). Moreover, the small number of participants might not have been enough to reach statistical significance.

Obesity is known to be a state of abnormal immune activity and increased susceptibility to inflammatory diseases, such as periodontal disease, diabetes, airway inflammation, cardiovascular disease and fatty liver disease [17, 35]. Therefore, we hypothesized that obesity might also be related to sialolithiasis. However, a comparison among the 5 groups divided by BMI did not show any difference. Additional analyses of these groups according to sex and age did not show any differences. There are no previous reports regarding obesity and sialolithiasis. One study reported that the salivary flow is not decreased in obese patients compared to nonobese patients. However, the activity of salivary enzymes, such as lipase and α-amylase, which break down nutrients, was much higher in the obese group [36]. Therefore, obesity can be considered the cause of chronic inflammation, which can lead to the formation of salivary stone, but a normal amount of salivary flow and enzymes with high activity can interfere with the salivary stone formation.

This study has several benefits. In contrast to hospital-based studies, we used participants from a large representative nationwide population who had undergone health screening examinations. We analyzed the ORs for tobacco smoking, alcohol consumption and obesity based on BMI in the sialolithiasis group and compared them to the ORs in a well-matched control group. The control group was randomly selected and matched by age group, sex, income group, region of residence, and medical history (e.g., hypertension, diabetes, and dyslipidemia) to prevent selection bias. Moreover, we used an adjusted logistic regression model to minimize confounders.

This study has several limitations. First, we used patient claim codes from the HIRA database to confirm which individuals were diagnosed with sialolithiasis. These data may indicate the incidence of sialolithiasis. Second, the participants self-reported their health status and habits, and self-reporting can be biased. Third, we could not evaluate the amount of alcohol consumed, which has an important effect on oral health. Fourth, we did not have data from patients who were younger than 40 years old because the health screening examinations were performed in individuals older than 40 years. Fifth, the number of participants with sialolithiasis was relatively small because of the low incidence rate of this disease. Sixth, the rate of smoking was statistically significant different between the sialolithiasis and control groups, but the difference was not large. Finally, we did not observe an association between female sex and smoking. The low rates of smoking in women compared to men might have affected this finding (S2 Table).

## Conclusions

Tobacco smoking showed a positive association with sialolithiasis in the population ≥ 40 years old. However, the association of alcohol consumption and obesity with sialolithiasis did not reach statistical significance.

## Supplementary information

**Additional file 1: Supplement 1** Smoking and alcohol consumption. **Supplement 2** The rates of smoking, alcohol consumption, and obesity according to age group and sex. **Supplement 3** Crude and adjusted odds ratios (95% confidence interval) for smoking, drinking alcohol, and obesity in patients with sialolithiasis.

**Additional file 2.** STROBE Statement—checklist of items that should be included in reports of observational studies

## Data Availability

The datasets analyzed during the current study are in the public domain and are freely available from http://nhiss.nhis.or.kr/ with permission.

## Abbreviations

OR: Odds ratio
CI: Confidence interval
NHIS-HEALS: National health insurance service-health screening cohort
HIRA: Health insurance review and assessment
BMI: Body mass index
WPRO: regional office for the western pacific
