# Supplementary material for: The association between sialolithiasis and smoking, alcohol drinking and obesity in Korea: a nested case-control study
Source: BMC Public Health. 2020 Apr 17;20:516. doi: 10.1186/s12889-020-08674-w (PMC7164270; doi:10.1186/s12889-020-08674-w)
Supplement: Supplementary file 1 — Additional file 1: Supplement 1 Smoking and alcohol consumption. Supplement 2 The rates of smoking, alcohol consumption, and obesity according to age group and sex. Supplement 3 Crude and adjusted odds ratios (95% confidence interval) for smoking, drinking alcohol, and obesity in patients with sialolithiasis. [file 12889_2020_8674_MOESM1_ESM.docx]

**Table 1** General characteristics of the participants

| Characteristics | | Participants | | |
| --- | --- | --- | --- | --- |
|  |  | Sialolithiasis (n, %) | Control (n, %) | P-value |
| Age (years) | |  |  | 1.000 |
|  | 40-44 | 36 (3.8) | 144 (3.8) |  |
|  | 45-49 | 135 (14.3) | 540 (14.3) |  |
|  | 50-54 | 215 (22.7) | 860 (22.7) |  |
|  | 55-59 | 172 (18.2) | 688 (18.2) |  |
|  | 60-64 | 144 (15.2) | 576 (15.2) |  |
|  | 65-69 | 102 (10.8) | 408 (10.8) |  |
|  | 70-74 | 88 (9.3) | 352 (9.3) |  |
|  | 75-79 | 33 (3.5) | 132 (3.5) |  |
|  | 80-84 | 19 (2.0) | 76 (2.0) |  |
|  | 85+ | 3 (0.3) | 12 (0.3) |  |
| Sex | |  |  | 1.000 |
|  | Male | 493 (52.1) | 1,972 (52.1) |  |
|  | Female | 454 (47.9) | 1,816 (47.9) |  |
| Income | |  |  | 1.000 |
|  | 1 (lowest) | 130 (13.7) | 520 (13.7) |  |
|  | 2 | 100 (10.6) | 400 (10.6) |  |
|  | 3 | 128 (13.5) | 512 (13.5) |  |
|  | 4 | 222 (23.4) | 888 (23.4) |  |
|  | 5 (highest) | 367 (38.8) | 1,468 (38.8) |  |
| Region of residence | |  |  | 1.000 |
|  | Urban | 408 (43.1) | 1,632 (43.1) |  |
|  | Rural | 539 (56.9) | 2,156 (56.9) |  |
| Hypertension | | 290 (30.6) | 1,160 (30.6) | 1.000 |
| Diabetes | | 117 (12.4) | 468 (12.4) | 1.000 |
| Dyslipidemia | | 105 (11.1) | 420 (11.1) | 1.000 |
| Obesity (BMI, kg/m^2^) | |  |  | 0.100 |
|  | < 18.5 | 13 (1.4) | 92 (2.4) |  |
|  | ≥ 18.5 to < 23 | 314 (33.2) | 1,272 (33.6) |  |
|  | ≥ 23 to < 25 | 280 (29.6) | 1,051 (27.7) |  |
|  | ≥ 25 to < 30 | 299 (31.6) | 1,250 (33.0) |  |
|  | ≥ 30 | 41 (4.3) | 123 (3.2) |  |
| Smoking | |  |  | 0.047* |
|  | Nonsmoker | 640 (67.6) | 2,685 (70.9) |  |
|  | Past or current smoker | 307 (32.4) | 1,103 (29.1) |  |
| Alcohol consumption | |  |  | 0.334 |
|  | < 1 time per week | 717 (75.7) | 2,810 (74.2) |  |
|  | ≥ 1 time per week | 230 (24.3) | 978 (25.8) |  |

BMI: body mass index, kg/m^2^

*Chi-square test. Significance at P < 0.05

**Table 2** Crude and adjusted odds ratios (95% confidence interval) for smoking, drinking alcohol, and obesity in sialolithiasis patients.

| Characteristics | | | Crude model | | Adjusted model | |
| --- | --- | --- | --- | --- | --- | --- |
|  | |  | Crude† | P-value | Adjusted†‡ | P-value |
| Smoking | | | 1.27 (1.05-1.53) | 0.016* | 1.31 (1.08-1.59) | 0.006* |
| Alcohol | | | 0.90 (0.75-1.09) | 0.281 | 0.86 (0.71-1.04) | 0.119 |
| Obesity (BMI, kg/m^2^) | | |  | 0.100 |  | 0.092 |
|  | < 18.5 | | 0.57 (0.31-1.03) | 0.063 | 0.56 (0.31-1.01) | 0.055 |
|  | ≥ 18.5 to < 23 | | 1.00 |  | 1.00 |  |
|  | ≥ 23 to < 25 | | 1.08 (0.90-1.30) | 0.395 | 1.09 (0.90-1.31) | 0.376 |
|  | ≥ 25 to < 30 | | 0.97 (0.81-1.17) | 0.757 | 0.98 (0.81-1.17) | 0.800 |
|  | ≥ 30 | | 1.36 (0.93-1.98) | 0.111 | 1.36 (0.93-1.98) | 0.119 |

* Conditional logistic regression analysis, significance at P < 0.05

† Conditional model for obesity, smoking status (current or past smoker compared to nonsmoker), and frequency of alcohol consumption (≥ 1 time per week compared to < 1 time per week).

‡ Adjusted model including obesity, smoking status and frequency of alcohol consumption.

**Table 3** Crude and adjusted odds ratios (95% confidence interval) for smoking, drinking alcohol, and obesity in sialolithiasis patients in each stratified group according age and sex

| Characteristics | | | Crude model | | Adjusted model | |
| --- | --- | --- | --- | --- | --- | --- |
|  | |  | Crude† | P-value | Adjusted†‡ | P-value |
| **< 60 years (n = 2,790)** | | | | | | |
| Smoking | | | 1.32 (1.03-1.69) | 0.027* | 1.36 (1.06-1.75) | 0.017* |
| Alcohol consumption | | | 0.95 (0.75-1.19) | 0.628 | 0.90 (0.71-1.13) | 0.352 |
| Obesity (BMI, kg/m^2^) | | |  | 0.164 |  | 0.164 |
|  | < 18.5 | | 0.65 (0.29-1.48) | 0.307 | 0.63 (0.28-1.43) | 0.269 |
|  | ≥ 18.5 to < 23 | | 1.00 |  | 1.00 |  |
|  | ≥ 23 to < 25 | | 0.96 (0.76-1.22) | 0.761 | 0.96 (0.76-1.22) | 0.748 |
|  | ≥ 25 to < 30 | | 0.87 (0.69-1.10) | 0.253 | 0.87 (0.69-1.11) | 0.256 |
|  | ≥ 30 | | 1.50 (0.95-2.39) | 0.084 | 1.49 (0.94-2.36) | 0.092 |
| **≥ 60 years (n = 1,945)** | | | | | | |
| Smoking | | | 1.19 (0.88-1.61) | 0.266 | 1.24 (0.91-1.69) | 0.168 |
| Alcohol consumption | | | 0.83 (0.61-1.14) | 0.244 | 0.80 (0.58-1.10) | 0.171 |
| Obesity (BMI, kg/m^2^) | | |  | 0.178 |  | 0.160 |
|  | < 18.5 | | 0.52 (0.22-1.24) | 0.141 | 0.52 (0.22-1.23) | 0.137 |
|  | ≥ 18.5 to < 23 | | 1.00 |  | 1.00 |  |
|  | ≥ 23 to < 25 | | 1.30 (0.97-1.75) | 0.079 | 1.32 (0.98-1.77) | 0.070 |
|  | ≥ 25 to < 30 | | 1.15 (0.86-1.54) | 0.340 | 1.16 (0.87-1.55) | 0.313 |
|  | ≥ 30 | | 1.11 (0.57-2.16) | 0.756 | 1.13 (0.58-2.20) | 0.724 |
| **Male (n = 2,645)** | | | | | | |
| Smoking | | | 1.24 (1.02-1.52) | 0.034* | 1.27 (1.03-1.56) | 0.023* |
| Alcohol consumption | | | 0.94 (0.77-1.14) | 0.514 | 0.89 (0.73-1.10) | 0.284 |
| Obesity (BMI, kg/m^2^) | | |  | 0.142 |  | 0.144 |
|  | < 18.5 | | 0.47 (0.21-1.07) | 0.071 | 0.47 (0.21-1.06) | 0.068 |
|  | ≥ 18.5 to < 23 | | 1.00 |  | 1.00 |  |
|  | ≥ 23 to < 25 | | 0.99 (0.77-1.28) | 0.940 | 0.99 (0.77-1.28) | 0.962 |
|  | ≥ 25 to < 30 | | 0.85 (0.66-1.10) | 0.221 | 0.86 (0.66-1.11) | 0.233 |
|  | ≥ 30 | | 1.38 (0.79-2.41) | 0.259 | 1.37 (0.79-2.40) | 0.265 |
| **Female (n = 2,270)** | | | | | | |
| Smoking | | | 1.47 (0.83-2.58) | 0.186 | 1.68 (0.94-3.00) | 0.082 |
| Alcohol consumption | | | 0.75 (0.47-1.21) | 0.243 | 0.69 (0.42-1.13) | 0.136 |
| Obesity (BMI, kg/m^2^) | | |  | 0.505 |  | 0.435 |
|  | < 18.5 | | 0.70 (0.29-1.69) | 0.427 | 0.66 (0.28-1.60) | 0.361 |
|  | ≥ 18.5 to < 23 | | 1.00 |  | 1.00 |  |
|  | ≥ 23 to < 25 | | 1.18 (0.91-1.54) | 0.220 | 1.19 (0.91-1.55) | 0.205 |
|  | ≥ 25 to < 30 | | 1.11 (0.86-1.45) | 0.421 | 1.13 (0.87-1.47) | 0.367 |
|  | ≥ 30 | | 1.36 (0.81-2.27) | 0.241 | 1.37 (0.82-2.30) | 0.225 |

* Conditional logistic regression analysis, significance at P < 0.05

† Conditional model including age, sex, income, region of residence, hypertension, diabetes, and dyslipidemia history.

‡ Model adjusted for obesity, smoking status (current or past smoker vs. nonsmoker), and frequency of alcohol consumption (≥ 1 time per week compared to < 1 time per week).

**Supplement 1** Smoking and alcohol consumption

| Characteristics | | Total participants | | |
| --- | --- | --- | --- | --- |
|  |  | Sialolithiasis (n, %) | Control (n, %) | P-value |
| Smoking status | |  |  | 0.139 |
|  | Nonsmoker | 640 (67.6) | 2,685 (70.9) |  |
|  | Past smoker | 122 (12.9) | 440 (11.6) |  |
|  | Current smoker | 185 (19.5) | 663 (17.5) |  |
| Duration of smoking (total) | |  |  | 0.123 |
|  | Nonsmoker | 640 (67.6) | 2,685 (70.9) |  |
|  | < 20 years | 90 (9.5) | 339 (8.9) |  |
|  | ≥ 20 years | 217 (22.9) | 764 (20.2) |  |
| Current cigarettes per day | |  |  | 0.279 |
|  | 0 cigarette per day | 762 (80.5) | 3,125 (82.5) |  |
|  | < 20 cigarettes per day | 123 (13.0) | 457 (12.1) |  |
|  | ≥ 20 cigarettes per day | 62 (6.5) | 206 (5.4) |  |
| Frequency of alcohol consumption | |  |  | 0.599 |
|  | Nondrinker | 578 (61.0) | 2,279 (60.2) |  |
|  | < 1 time per week | 139 (14.7) | 531 (14.0) |  |
|  | ≥ 1 time per week | 230 (24.3) | 978 (25.8) |  |
| Amount of alcohol consumed at a time | |  |  | 0.425 |
|  | < 1 bottle of soju | 727 (76.8) | 2,861 (75.5) |  |
|  | ≥ 1 bottle of soju | 220 (23.2) | 927 (24.5) |  |

*Chi-square test. Significance at P < 0.05

Current smoking status was classified as nonsmoker, past smoker, and current smoker. We merged smokers and past smokers into one group (current or past smokers, Table 1).

The duration of smoking was classified as nonsmoker, < 5 years, 5-9 years, 10-19 years, 20-29 years, and ≥ 30 years. It was recategorized as nonsmoker, < 20 years, and ≥ 20 years.

Current cigarettes smoked per day was classified as nonsmoker, < 10 cigarettes, 10-19 cigarettes, 20-39 cigarettes, and ≥ 40 cigarettes. It was recategorized as 0 cigarettes per day, < 20 cigarettes per day, and ≥ 20 cigarettes per day.

Frequency of alcohol consumption was categorized as nondrinker, 2-3 times per month, 1-2 times per week, 3-4 times per week, and ≥ 5 times per week. It was recategorized as nondrinker, < 1 time per week, and ≥ 1 time per week. We merged nondrinkers and < 1 time per week drinkers into one group (< 1 time per week, Table 1).

The amount of alcohol consumed at a time was classified as < 1 bottle of soju, ~ 1 bottle of soju, > 1 to < 2 bottle of soju, and ≥ 2 bottle of soju. It was recategorized as < 1 bottle of soju and ≥ 1 bottle of soju. Soju is the most common alcoholic beverage in Korea. Generally, a bottle of soju contains 17.5% alcohol per 360 ml. A bottle of soju is equivalent to approximately 3.5 bottles of beer.

**Supplement 2** The rates of smoking, alcohol consumption, and obesity according to age group and sex.

| Characteristics | | Age group | | | Sex | | |
| --- | --- | --- | --- | --- | --- | --- | --- |
|  | | <60 years | ≥60 years | P-value | Male | Female | P-value |
| Smoking status | |  |  | <0.001* |  |  | <0.001* |
|  | Nonsmoker | 1,879 (67.3) | 1,446 (74.3) |  | 1,119 (45.4) | 2,206 (97.2) |  |
|  | Past or current smoker | 911 (32.7) | 499 (25.7) |  | 1,346 (54.6) | 64 (2.8) |  |
| Frequency of alcohol consumption | |  |  | <0.001* |  |  | <0.001* |
|  | < 1 time per week | 1,979 (70.9) | 1,548 (79.6) |  | 1,388 (56.3) | 2,139 (94.2) |  |
|  | ≥ 1 time per week | 811 (29.1) | 397 (20.4) |  | 1,077 (43.7) | 131 (5.8) |  |
| Obesity (BMI, kg/m^2^) | |  |  | 0.002* |  |  | <0.001* |
|  | < 18.5 | 48 (1.7) | 57 (2.9) |  | 62 (2.5) | 43 (1.9) |  |
|  | ≥ 18.5 to < 23 | 978 (35.1) | 608 (31.3) |  | 729 (29.6) | 857 (37.8) |  |
|  | ≥ 23 to < 25 | 782 (28.0) | 549 (28.2) |  | 754 (30.6) | 577 (25.4) |  |
|  | ≥ 25 to < 30 | 879 (31.5) | 670 (34.4) |  | 849 (34.4) | 700 (30.8) |  |
|  | ≥ 30 | 103 (3.7) | 61 (3.1) |  | 71 (2.9) | 93 (4.1) |  |

*Chi-square test. Significance at P < 0.05

**Supplement 3** Crude and adjusted odds ratios (95% confidence interval) for smoking, drinking alcohol, and obesity in patients with sialolithiasis.

| Characteristics | | | Crude model | | Adjusted model | |
| --- | --- | --- | --- | --- | --- | --- |
|  | |  | Crude† | P-value | Adjusted†‡ | P-value |
| Smoking | | |  |  |  |  |
|  | Nonsmoker | | 1.00 |  | 1.00 |  |
|  | < 20 years | | 1.11 (0.87-1.43) | 0.394 | 1.24 (0.94-1.63) | 0.123 |
|  | ≥ 20 years | | 1.19 (1.00-1.42) | 0.048* | 1.34 (1.08-1.66) | 0.007* |
| Alcohol | | |  |  |  |  |
|  | < 1 bottle of soju | | 1.00 |  | 1.00 |  |
|  | ≥ 1 bottle of soju | | 0.93 (0.79-1.11) | 0.425 | 0.86 (0.70-1.04) | 0.125 |
| Obesity (BMI, kg/m^2^) | | |  |  |  |  |
|  | < 23 | | 1.00 |  | 1.00 |  |
|  | ≥ 23 | | 1.07 (0.92-1.24) | 0.396 | 1.08 (0.93-1.26) | 0.313 |

* Conditional logistic regression analysis, significance at P < 0.05

† Conditional model for obesity, duration of smoking (nonsmoker, < 20 years, and ≥ 20 years), and amount of alcohol (< 1 bottle of soju and ≥ 1 bottle of soju).

‡ Adjusted model including obesity, smoking duration, amount of alcohol consumed, and obesity.
